# *Astragali Radix-Coptis Rhizoma* Herb Pair Attenuates Atherosclerosis in ApoE-/- Mice by Regulating the M1/M2 and Th1/Th2 Immune Balance and Activating the STAT6 Signaling Pathway

**DOI:** 10.1155/2022/7421265

**Published:** 2022-02-07

**Authors:** Zhaoyu Li, Dufang Ma, Yongcheng Wang, Sijia Wu, Lin Wang, Yuehua Jiang, Yongjian Zhang, Xiao Li

**Affiliations:** ^1^First Clinical Medical College, Shandong University of Traditional Chinese Medicine, Jinan 250014, China; ^2^Department of Cardiovascular, Affiliated Hospital of Shandong University of Traditional Chinese Medicine, Jinan 250011, China; ^3^College of Traditional Chinese Medicine, Shandong University of Traditional Chinese Medicine, Jinan 250014, China; ^4^Central Laboratory, Affiliated Hospital of Shandong University of Traditional Chinese Medicine, Jinan 250011, China

## Abstract

**Objective:**

Immune imbalance and the inflammatory response are associated with atherosclerosis (AS) progression. *Astragali Radix* and *Coptis Rhizoma* (ARCR) are an ancient and classic herb pair that is used in herbal medicines for the treatment of coronary heart disease. We focused on the effects and mechanisms of the ARCR herb pair attenuation of atherosclerosis in apolipoprotein E knockout (ApoE-/-) mice.

**Methods:**

ApoE-/- mice were fed a high-fat diet for 12 weeks to establish a model of AS. The ApoE-/- mice were randomly divided into a model group, simvastatin group (Simva), *Astragali Radix* group (AR), *Coptis Rhizoma* group (CR), *Astragali Radix-Coptis Rhizoma* group (ARCR), and *Astragali Radix-Coptis Rhizoma* + signal transducer and activator of transcription factor 6 (STAT6) inhibitor (AS1517499) group (ARCR + AS1517499). C57BL/6 mice were used as controls. Each group was administered the corresponding drugs, and mice in the model and control groups were given the same volume of normal saline once daily for 6 weeks. The body weights of the mice were observed regularly. The effect of the ARCR herb pair on lipid content in peripheral blood was evaluated using blood lipid tests. The levels of serum matrix metalloproteinase-9 (MMP-9), interleukins-12 (IL-12), IL-10, interferon-*γ* (IFN-*γ*), and IL-4 were determined to assess inflammation. Oil Red O staining, Sirius Red staining, and immunohistochemistry were used to observe changes in plaque stability. Western blotting was used to assay M1/M2 macrophages, Th1/Th2 cells, and STAT6 signaling pathway protein expression. Flow cytometry and immunofluorescence were used to detect M1/M2 macrophages and Th1/Th2 cells and reflect the immune imbalance.

**Results:**

The ARCR herb pair significantly reduced blood lipids levels and plaque vulnerability and regulated the levels of inflammatory factors and the number of M1/M2 macrophages and Th1/Th2 cells in ApoE-/- AS mice. It also decreased iNOS and T-bet protein levels and increased the Arg-1 and GATA-3 protein levels. The ARCR herb pair also increased STAT6 phosphorylation. A STAT6 inhibitor attenuated the regulation of M1/M2 and Th1/Th2 markers induced by the ARCR herb pair.

**Conclusion:**

The ARCR herb pair regulates blood lipid metabolism and attenuates atherosclerosis via regulation of M1/M2 and Th1/Th2 immune balance, which is achieved partially by increasing STAT6 phosphorylation. Our study provides new evidence for the possible use of ARCR herb pair in the prevention and treatment of AS.

## 1. Introduction

Atherosclerosis (AS) is a chronic inflammatory disease, and immune cells play an important role in the pathological process. Innate and adaptive immunity are involved in the occurrence and development of AS [1, 2]. The ratio of proinflammatory/anti-inflammatory immune cells positively correlates with the severity of AS [3]. The polarization classification of immune cells in AS and the regulation of inflammatory balance are highly significant for the prevention and control of AS and other related cardiovascular diseases [4–6]. With the deepening of AS immune-inflammatory research, the immune regulation mechanism has become an important target for improving AS. Current AS immunotherapy focuses on anti-inflammatory agents, such as statins, which play an anti-inflammatory effect while reducing blood lipid. A pre-protein converting enzyme subtilisin 9 (PCSK9) inhibitor recently entered the clinic as a new choice for lipid-lowering therapy. This inhibitor significantly reduced LDL levels and further reduced the risk of cardiovascular events in patients with atherosclerotic cardiovascular disease. However, it unilaterally inhibited proinflammatory factors in arterial plaques, and long-term application reduced the body's defense function [7]. Therefore, clinical treatment should not unilaterally be anti-inflammatory, but it should adjust the balance of pro- and anti-inflammatory factors, with the goal of restoring immune balance. Treatments that target and regulate the immune balance of AS should be investigated and developed.

The recruitment of immune cells is the main factor that induces AS. The inflammatory reaction caused by the abnormal balance between pro- and anti-inflammatory immune cells promotes the occurrence and development of AS. Imbalance in the number and function of immune cells plays an important role in the formation, development, and rupture of AS, including innate immunity with mononuclear-macrophage polarized M1 (proinflammatory) and M2 (anti-inflammatory) cells as the core inflammation. The adaptive immune-inflammatory response is primarily manifested as the proinflammatory/anti-inflammatory imbalance of helper T cells (Th cells). The balance between the pro-AS inflammatory response and the anti-AS anti-inflammatory response is regulated by the interaction of these two major immune systems and related cytokines and mediators [8]. The number of proinflammatory cells and the inflammatory response are often increased under the pathological conditions of AS and accompanied by a decrease in anti-inflammatory cells, which creates an imbalance in the immune-inflammatory response. The ratio of proinflammatory/anti-inflammatory immune cells positively correlates with the severity of AS [3]. Accordingly, we demonstrated that regulating the imbalance of immune cells may improve plaque stability and delay the progression of AS [9, 10]. JAK/STAT is a transmembrane signal transduction pathway, which plays an important role in the proliferation and differentiation of immune cells [11]. There are many subtypes of STAT that have different roles in regulating the differentiation of macrophages and T lymphocytes [12, 13]. Among them, STAT6 signal activation induces the differentiation of M2 macrophage and Th2 lymphocyte. Therefore, activating the STAT6 signaling pathway could regulate the balance of M1/M2 macrophage and Th1/Th2 lymphocyte and inhibit inflammation.

*Astragali Radix-Coptis Rhizoma* (ARCR) is commonly used in traditional Chinese herbal medicine. *Astragali Radix* reinforces qi and invigorates vitality, and *Coptis Rhizoma* clears heat and detoxifies. The combination of these two herbs invigorates qi, detoxifies, and regulates blood circulation, and it is often used for the treatment of cardiovascular diseases. *Astragali Radix* and *Coptis Rhizoma* also have anti-inflammatory, immune-regulating, and antiatherosclerotic effects [14–16]. However, its effect and detailed mechanisms on atherosclerosis need further investigation. Considering the important role of M1/M2 macrophage and Th1/Th2 cell balance in inflammation and atherosclerosis, we hypothesized that the ARCR herb pair protected against atherosclerosis by regulating the M1/M2 and Th1/Th2 immune balance via activation of the STAT6 signaling pathway.

To test this hypothesis, we validated the antiatherosclerosis effect in atherosclerotic apolipoprotein E knockout (ApoE-/-) mice treated with the ARCR herb pair. We used a signal transducer and activator of transcription factor 6 (STAT6) pathway inhibitor to identify a possible pathway and explore the underlying mechanism of the immune imbalance in mice with AS. The data suggested that the ARCR herb pair attenuated atherosclerosis in ApoE-/- mice by regulating M1/M2 and Th1/Th2 immune balance via activation of the STAT6 signaling pathway.

## 2. Materials and Methods

### 2.1. Animal Groups and Drug Administration

Male ApoE-/- mice (8 weeks old) and male C57BL/6 mice (8 weeks old) were obtained from Beijing Vital River Laboratory Animal Technology Co., Ltd. (Beijing, China). All procedures were performed strictly in accordance with the Guidelines for the Care and Use of Laboratory Animals issued by the Ministry of Science and Technology of China and approved by the Institutional Animal Care and Use Committee of Shandong University of Traditional Chinese Medicine (NO. AWE-2019-039). The mice were acclimated for 1 week. The ApoE-/- mice were fed a high-fat diet (fat: 15%, cholesterol: 1.25%), and C57BL/6 mice were fed ordinary rodent chow for 12 weeks (Table 1). The project was divided into two parts. Experiment 1 evaluated the effects of ARCR herbs on atherosclerosis in ApoE-/- mice via immunomodulation after administration. Experiment 2 assessed the effects of STAT6 inhibitor treatment to explore the pharmacological mechanism. The ApoE-/- mice in Experiment 1 were randomly divided into five groups (*n* = 10): model group (high-fat diet), simvastatin group (high-fat diet and simvastatin 5 mg/kg/day), *Astragali Radix* group (high-fat diet and *Astragali Radix* 3.9 g/kg/day), *Coptis Rhizoma* group (high-fat diet and *Coptis Rhizoma* 1.3 g/kg/day), and *Astragali Radix-Coptis Rhizoma* group (high-fat diet and *Astragali Radix-Coptis Rhizoma* 5.2 g/kg/day). Ten C57BL/6 mice were included as the control group (fed ordinary rodent chow). ApoE-/- mice in Experiment 2 were grouped as follows (*n* = 10): model group, *Astragali Radix-Coptis Rhizoma* group, and *Astragali Radix-Coptis Rhizoma* + STAT6 inhibitor (AS1517499) group (high-fat diet and *Astragali Radix-Coptis Rhizoma* 5.2 g/kg/day and intraperitoneally injected with AS1517499 10 mg/kg/day). Ten C57BL/6 mice were used as the control group. Mice went through gavage once daily for 6 weeks.

### 2.2. Drug Preparation

The doses of *Astragali Radix* and *Coptis Rhizoma* for one adult per day were used. *Astragali Radix* (30 g) and *Coptis Rhizoma* (10 g) were purchased from the affiliated hospital of Shandong University of Traditional Chinese Medicine, and herbs were identified by Prof. Chuanjiang Ma of TCM Pharmacy. The herbs were mixed and extracted under reflux with distilled water (1 : 10 volumes) twice for 1 h each. The solution was combined and concentrated to a relative density of 1.20–1.25 (70–80°C). Raw herbs (2 g granules/g) were prepared to obtain granules. The solution was 0.5 g raw medicinal herbs/mL.

### 2.3. Body Weights

The body weights of mice were measured every Monday from 9 am to 10 am.

### 2.4. Detection of Serum Lipids

After treatment, each mouse was euthanized via cervical dislocation under anaesthesia induced by an intraperitoneal injection of 4% sodium pentobarbital. Blood samples collected from the retroorbital plexus in mice were placed for 2 h at room temperature, followed by centrifugation at 3000 rpm for 5 min at 4°C. Total cholesterol (TC) and triglycerides (TG) in mouse serum were detected using relevant kits (CCHUILI).

### 2.5. ELISAs

Serum matrix metalloproteinase-9 (MMP-9) and inflammatory factors, including interleukins-12 (IL-12), IL-10, interferon-*γ* (IFN-*γ*), and IL-4, were measured using the following ELISA kits (Elabscience): Mouse MMP-9 ELISA kit (Cat No. E-EL-M3052), Mouse IL-12 ELISA kit (Cat No. E-EL-M0726c), Mouse IL-10 ELISA kit (Cat No. E-EL-M0046c), Mouse IFN-*γ* ELISA kit (Cat No. E-EL-M0048c), and Mouse IL-4 ELISA kit (Cat No. E-EL-M0043c). All procedures followed the manufacturer's instructions.

### 2.6. Immunofluorescence and Immunohistochemistry

Hearts with attached aortic roots were harvested, quickly frozen in OCT, and cut into sections at a thickness of 5 *μ*m. Further immunohistochemical and immunofluorescent analyses were performed as described below. Mouse aortic sinus cryosections were stained with anti-inducible nitric oxide synthase antibody [iNOS] (Proteintech, Cat No. 18985-1-AP), anti-arginase-1 antibody [Arg-1] (Proteintech, Cat No. 16001-1-AP), anti-CD4 antibody [CD4] (CST, Cat No. 96127S), anti-T box expressed in T cell antibody [T-bet] (Proteintech, Cat No. 13700-1-AP), anti-GATA binding protein-3 antibody [GATA-3] (Proteintech, Cat No. 66400-1-Ig), anti-alpha smooth muscle actin antibody [SMA] (Servicebio, Cat No. GB111364), and anti-monocyte + macrophage antibody [MOMA-2] (Abcam, Cat No. ab33451). Plaque composition was assessed in cross sections of aortic root by immunostaining for MOMA-2 (macrophages) and *α*-smooth muscle actin (smooth muscle cells). Sirius Red staining was used for collagen, and Oil Red O was used for lipid contents. The collagen content of lesions was assessed using Sirius Red-stained slides under polarizing light. Images were acquired on an inverted digital microscope system and processed using Image-Pro Plus 6.0. For each slide, at least three images were captured and evaluated in a blinded manner. The percentage of positively colored area of the lesion area was calculated for each mouse. The plaque vulnerability index was calculated using the formula: vulnerability index = (lipid deposit % + macrophages %)/(collagen fibers % + SMCs %) [17].

### 2.7. Western Blot Analysis

Protein was extracted from the aortas. The tissues were homogenized in ice-cold RIPA buffer, and protein concentrations were determined using an enhanced BCA protein assay kit (Beyotime Biotechnology, China). Forty micrograms of protein was electrophoretically separated using SDS-PAGE and transferred to PVDF membranes for blotting. The membranes were blocked with 5% nonfat milk in TBST for 1 hour at room temperature and incubated overnight at 4°C with primary antibodies against iNOS (Proteintech, Cat No. 18985-1-AP, 1 : 2000), Arg-1 (Proteintech, Cat No. 16001-1-AP, 1 : 5000), T-bet (Proteintech, Cat No. 13700-1-AP, 1 : 800), GATA-3 (Proteintech, Cat No. 66400-1-Ig, 1 : 3000), STAT6 (Servicebio, Cat No. GB111381, 1 : 1000), p-STAT6 [EPR22599-78] (Abcam, Cat No.ab263947, 1 : 1000), and *β*-actin (Proteintech, Cat No. 20536-1-AP, 1 : 2000). After the membrane was washed in TBST five times for five minutes each time, the blots were incubated for 1 h with goat anti-rabbit IgG (Servicebio, Cat No. GB23303, 1 : 20000) or goat anti-mouse IgG (Servicebio, Cat No. GB23301, 1 : 20000). The optical density intensity of each band was measured using FluorChem Q 3.4 (ProteinSimple, USA) [18].

### 2.8. Flow Cytometry

The mouse spleen was removed, and a red blood cell lysis solution and PBS were added dropwise for grinding. The grinding solution was aspirated, and a single cell suspension was screened at a cell concentration of 1–2×10^6^ cells/mL. The stained cells were acquired using an Agilent NovoCyte (Agilent Technologies, USA) and analyzed with FlowJo v9.0 software. Antibody colocations were performed as follows:

- F4/80 + CD11c+ M1 macrophages: PE-anti-mouse F4/80 antibody (Bioscience, Cat No. 12-4801-80) and FITC-anti-mouse CD11c antibody (Biolegend, Cat No. 117305);
- F4/80 + CD206+ M2 macrophages: PE-anti-mouse F4/80 antibody (Bioscience, Cat No. 12-4801-80) and PerCP-anti-mouse CD206 (MMR) antibody (Biolegend, Cat No. 141715);
- CD4 + CD25 + T-bet+ Th1 cells: FITC-anti-mouse CD4 antibody (Biolegend, Cat No. 100405), PerCP-anti-mouse CD25 antibody (Bioscience, Cat No. 45-0251-80) and PE-anti-T-bet antibody (Bioscience, Cat No. 12-5825-80); and
- CD4 + CD25 + GATA-3+ Th2 cells: FITC-anti-mouse CD4 antibody (Biolegend, Cat No. 100405), PerCP-anti-mouse CD25 antibody (Bioscience, Cat No. 45-0251-80) and PerCP-anti-GATA-3 antibody (Bioscience, Cat No. 46-9966-42).

### 2.9. Statistical Analysis

Statistical analyses of the data were performed using SPSS 26.0 software. Quantitative data are presented as the means ± SD and were analyzed using single-factor analysis of variance (ANOVA) followed by Dunnett's test or the Student-Newman–Keuls test. *P* < 0.05 was considered statistically significant.

## 3. Results

### 3.1. *Astragali Radix-Coptis Rhizoma* Herb Pair Improved Body Weight and Blood Lipids

**Figure 1:**
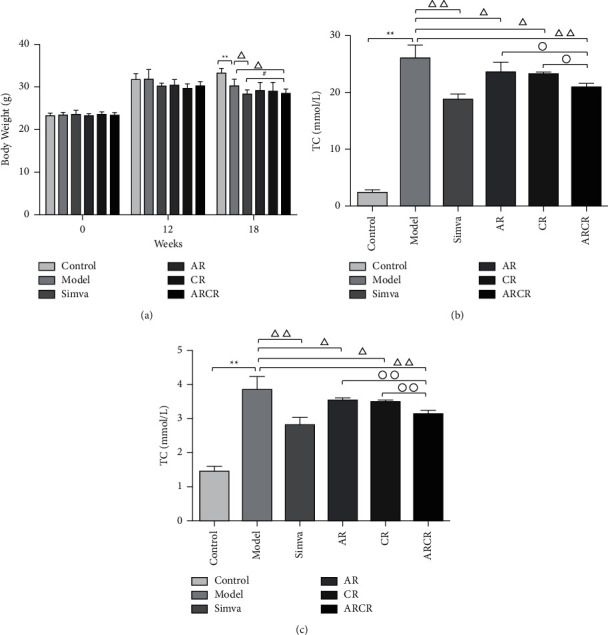
Evaluation of body weight and blood lipids. (a) Body weight, (b) TC, and (c) TG were measured in different groups. The data are presented as the means ± SD, *n* = 10. ^*∗*^*P* < 0.05, ^*∗∗*^*P* < 0.01 vs. the control group; ^Δ^*P* < 0.05, ^ΔΔ^*P* < 0.01 vs. the model group; °*P* < 0.05, ^∘∘^*P* < 0.01 vs. ARCR group; ^#^*P* < 0.05 vs. Simva group.

The body weight of the C57BL/6 mice was much heavier than the ApoE-/- mice at the end of the 18th week (*P* < 0.01). Compared to the model group, the body weight of mice in each drug intervention group showed less gain during 18 weeks, and the ARCR group showed a more significant reduction in body weight at the end of the 18th week than the AR or CR group. There was no significant difference with the Simva group (*P* > 0.05), which suggests an improvement effect of ARCR herb pair on the physical condition (Figure 1(a)).

Hyperlipidemia induces AS. Therefore, we tested the levels of serum lipids in ApoE-/- mice. Compared to a normal diet, a high-fat diet elevated TC and TG (*P* < 0.01). Compared to the model group, the TC and TG contents of the Simva group and ARCR group were significantly reduced (*P* < 0.01). The ARCR group had a better reducing effect than the AR group and CR group (*P* < 0.01 or *P* < 0.05, Figures 1(b) and 1(c)).

### 3.2. *Astragali Radix-Coptis Rhizoma* Herb Pair Increased Plaque Stability

**Figure 2:**
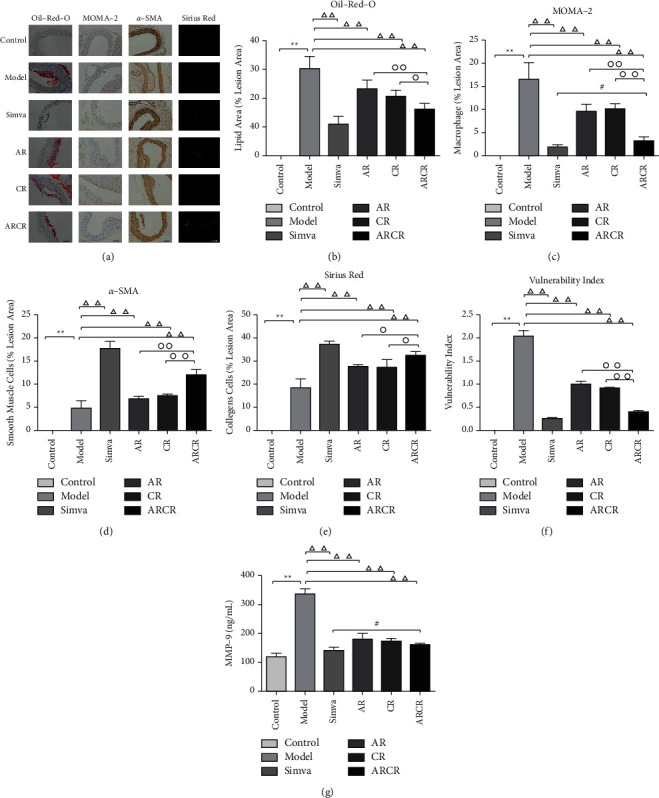
The ARCR herb pair significantly increased AS plaque stability in atherosclerotic ApoE-/- mice. (a) Oil red O staining of lipids, immunohistochemical staining of MOMA-2 (macrophage marker) and smooth muscle cells, and Sirius red staining of collagen. (b) Lipid area of oil red O staining. (c-d) Macrophages and smooth muscle cells were assessed using immunohistochemical staining. (e) Collagen of Sirius red staining. (f) Vulnerability index of plaque in corresponding groups calculated as (lipid deposit% + macrophages%)/(collagen fibers% + smooth muscle cells%). (g) ELISA for the expression of MMP-9. The data are presented as the means ± SD, *n* = 10. ^*∗*^*P* < 0.05, ^*∗∗*^*P* < 0.01 vs. the control group; ^Δ^*P* < 0.05, ^ΔΔ^*P* < 0.01 vs. the model group; °*P* < 0.05, ^∘∘^*P* < 0.01 vs. ARCR group; ^#^*P* > 0.05 vs. Simva group. (*n* = 6, scale bars: 50 *μ*m).

Atherosclerotic vulnerable plaques are characterized by a mass of macrophage infiltration and lipid accumulation, and a thin cap with less collagen and smooth muscle cells. As shown in Figure 2, the morphology of plaques in the model group was consistent with the features of typical vulnerable plaques (*P* < 0.01). The composition of plaques changed obviously after drug treatment, especially in the Simva group and ARCR group, in which the contents of lipids and macrophages decreased significantly, but collagen and smooth muscle cells increased significantly. Therefore, the vulnerability index [vulnerability index = (lipid deposit % + macrophages %)/(collagen fibers % + SMCs %)] of plaques in drug-treated mice was significantly reduced compared to the model group (*P* < 0.01). The ARCR group was superior to the AR and CR groups (*P* < 0.01) but not as good as the Simva group. These results indicated that the ARCR herb pair enhanced atherosclerotic plaque stability.

MMP-9 is closely related to plaque stability. As shown in Figure 2(g), the content of mice in the model group was significantly higher than the control group (*P* < 0.01). Compared to the model group, the ARCR group had a significantly reduced level of MMP-9 (*P* < 0.01). Comparison of the three groups of Chinese medicines showed that the ARCR group had the lowest MMP-9, but there was no significant difference from the AR and CR groups (*P* > 0.05).

### 3.3. *Astragali Radix-Coptis Rhizoma* Herb Pair Regulated Serum Inflammatory Cytokines

**Figure 3:**
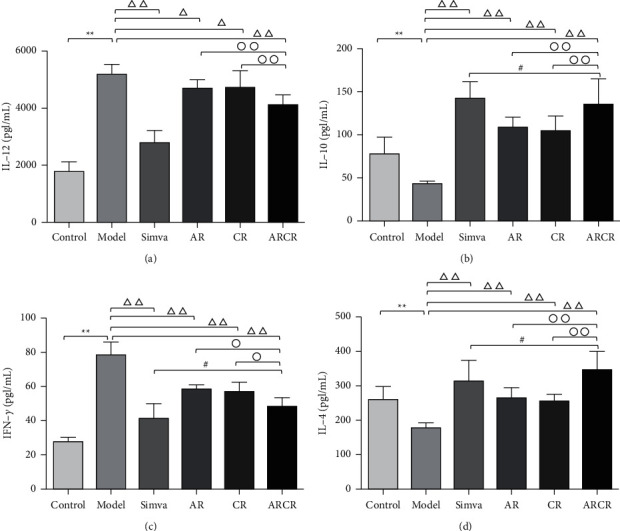
The ARCR herb pair regulated serum inflammatory cytokines. (a–d) ELISA for the expression of the proinflammatory cytokine IL-12, IFN-*γ* and the anti-inflammatory cytokines IL-10 and IL-4 in the supernatant. The data are presented as the means ± SD, *n* = 10. ^*∗*^*P* < 0.05, ^*∗∗*^*P* < 0.01 vs. the control group; ^Δ^*P* < 0.05, ^ΔΔ^*P* < 0.01 vs. the model group; °*P* < 0.05, ^∘∘^*P* < 0.01 vs. ARCR group; ^#^*P* > 0.05 vs. Simva group.

IL-12 and IL-10 are immunoregulatory factors secreted by M1/M2 macrophages that have pro- and anti-inflammatory effects, respectively. IFN-*γ* and IL-4 are pro- and anti-inflammatory factors secreted by Th1/Th2 cells. The results showed that IL-12 and IFN-*γ* in the model group were significantly increased compared to the control group (*P* < 0.01), and the ARCR group decreased significantly compared to the model group (*P* < 0.01, Figures 3(a) and 3(c)). In contrast, reduced secretion of the M2-inducing cytokine IL-10 and the Th2-inducing cytokine IL-4 was detected in the model group (*P* < 0.01), which were restored by the ARCR herb pair but not in the AR or CR group (*P* < 0.01 or *P* < 0.05, Figures 3(b) and 3(d)).

### 3.4. *Astragali Radix-Coptis Rhizoma* Herb Pair Regulated M1/M2 and Th1/Th2 Balance

**Figure 4:**
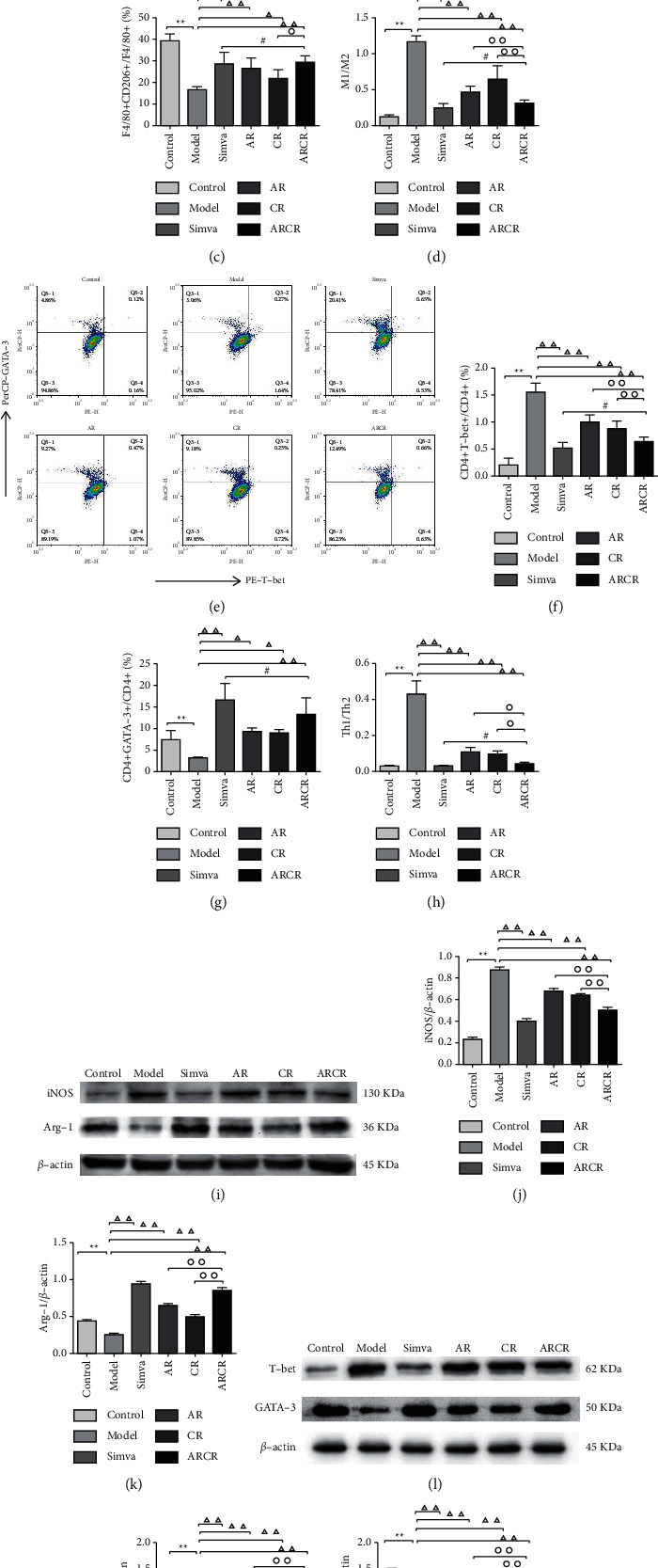
The ARCR herb pair regulated the M1/M2 and Th1/Th2 balance. (a) The expression of F4/80 + CD11c+ and F4/80 + CD206+ cells was examined using flow cytometry. (b–d) Percentages of M1 and M2 macrophages and M1/M2 macrophages. (e) The expression of CD4 + CD25 + T-bet+ and CD4 + CD25 + GATA-3+ cells was examined. (f–h) Percentages of Th1 and Th2 cells and Th1/Th2. (i) Representative Western blot of iNOS (M1 marker) and Arg-1 (M2 marker) in aortas. (j-k) iNOS and Arg-1 expression relative to the *β*-actin level. (l) Representative Western blot of T-bet (Th1 marker) and GATA-3 (Th2 marker) in aortas. (m-n) T-bet and GATA-3 expression relative to the *β*-actin level. The data are presented as the means ± SD, *n* = 4. ^*∗*^*P* < 0.05, ^*∗∗*^*P* < 0.01 vs. the control group; ^Δ^*P* < 0.05, ^ΔΔ^*P* < 0.01 vs. the model group; °*P* < 0.05, ^∘∘^*P* < 0.01 vs. ARCR group; ^#^*P* > 0.05 vs. Simva group.

On the basis of the modulated levels of serum cytokines, we hypothesized that the ARCR herb pair affected the differentiation of M1/M2 macrophages and Th1/Th2 cells in AS. Therefore, we used the mouse spleen for flow cytometry and the mouse aorta for Western blot and immunofluorescence detection of M1/M2 macrophages and Th1/Th2 cells. Figures 4(a) and 4(e) show the results of flow cytometry, and we confirmed elevated levels of F4/80 + CD11c+ M1 macrophages and CD4 + CD25 + T-bet + Th1 cells in the model group were strongly further increased compared to the control group (*P* < 0.01). The ARCR group significantly reduced the proportion of M1 and Th1 cells (*P* < 0.01, Figures 4(b) and 4(f)). F4/80 + CD206 + M2 macrophages and CD4 + CD25 + GATA-3 + Th2 cells were decreased in the model group mice in feedback to the increased M1 and Th1 (*P* < 0.01 or *P* < 0.05, Figures 4(c) and 4(g)). However, the ratio of M1/M2 and Th1/Th2 remained out of balance, which was significantly reversed by the ARCR herb pair but not AR or CR (*P* < 0.01, Figures 4(d) and 4(h)).

Western blot analysis (Figures 4(i)–4(n)) showed that the protein content of the M1 marker protein iNOS and the Th1 marker protein T-bet increased significantly in the model group compared to the control group. These changes were accompanied by a decrease in the protein content of the M2 marker protein Arg-1 and the Th2 marker protein GATA-3 (*P* < 0.01). The protein expression of iNOS and T-bet decreased after the intervention, and Arg-1 and GATA-3 increased. The ARCR herb pair had better effects than the AR or CR group (*P* < 0.01).

**Figure 5:**
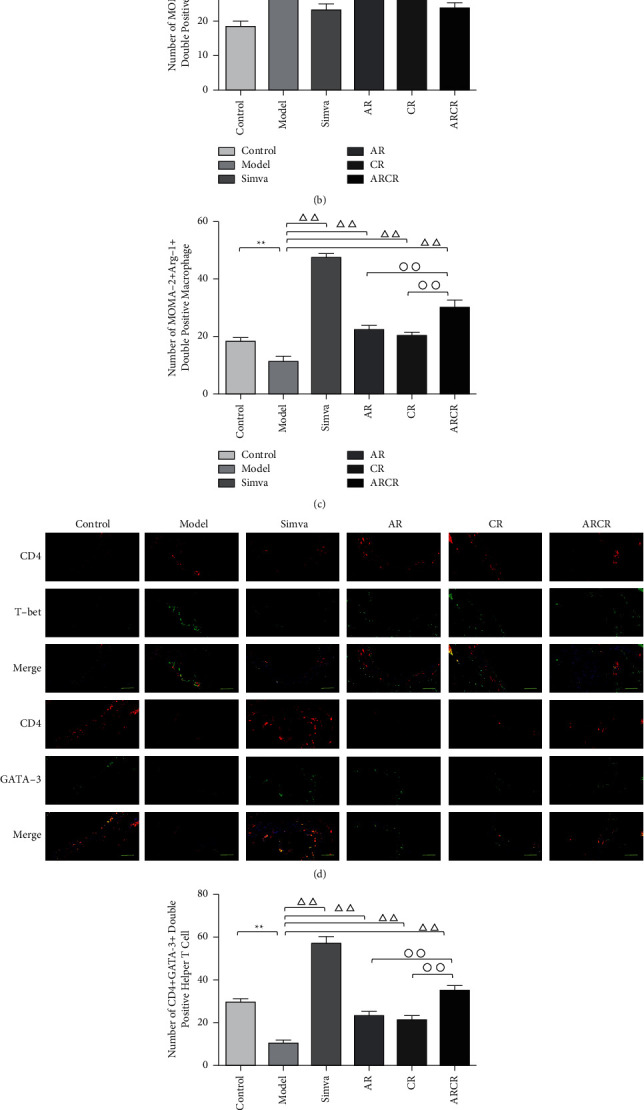
Effects of the ARCR herb pair on M1/M2 and Th1/Th2 balance in atherosclerotic lesions. (a) Representative images of MOMA-2 + iNOS+ and MOMA-2 + Arg-1+ macrophages (scale bars: 50 *μ*m). (b-c) Statistics of the number of MOMA-2 + iNOS+ and MOMA-2 + Arg-1+ macrophages in atherosclerotic lesions. (d) Representative images of CD4 + T-bet+ and CD4 + GATA-3+ cells (scale bars: 50 *μ*m). (e-f) Statistics of the number of CD4 + T-bet+ and CD4 + GATA-3+ cells in atherosclerotic lesions. The data are presented as the means ± SD, *n* = 3. ^*∗*^*P* < 0.05, ^*∗∗*^*P* < 0.01 vs. the control group; ^Δ^*P* < 0.05, ^ΔΔ^*P* < 0.01 vs. the model group; °*P* < 0.05, ^∘∘^*P* < 0.01 vs. ARCR group; ^#^*P* > 0.05 vs. Simva group.

Similar results were observed in the double immunofluorescence analysis. MOMA-2 + iNOS+ and MOMA-2 + Arg-1+ expression was used to identify and evaluate M1 and M2 macrophages, respectively (Figure 5(a)). CD4 + T-bet+ and CD4 + GATA-3+ represent Th1 and Th2 cells in aortic root cryosections (Figure 5(d)). The number of MOMA-2 + iNOS + double-positive macrophages (M1 macrophages) and CD4 + T-bet + double-positive cells (Th1 cells) in atherosclerotic lesions was significantly decreased in the drug intervention group compared to the model group (*P* < 0.01), and the number in the ARCR group was better than the AR or CR group (*P* < 0.01 or *P* < 0.05, Figures 5(b) and 5(e)). The number of MOMA-2 + Arg-1+ double-positive macrophages (M2 macrophages) and CD4 + GATA-3+ double-positive cells (Th2 cells) were significantly increased in the atherosclerotic lesions of the ARCR group (Figures 5(c) and 5(f)). These results suggested that ARCR herb pair treatment regulated M1/M2 and Th1/Th2 balance in atherosclerotic lesions.

### 3.5. *Astragali Radix-Coptis Rhizoma* Herb Pair Attenuated Atherosclerosis via a STAT6-Dependent Pathway

Previous studies demonstrated that activation of STAT6 induced M2 macrophage polarization and simultaneously induced T lymphocytes to differentiate into Th2-type cells and inhibited Th1-type cells [19, 20]. To investigate whether these inhibitory effects of the ARCR herb pair on atherosclerosis were exerted via activation of the STAT6 pathway, we designed the second part of the animal experiment, and an ARCR + STAT6 inhibitor (AS1517499) group was established.

**Figure 6:**
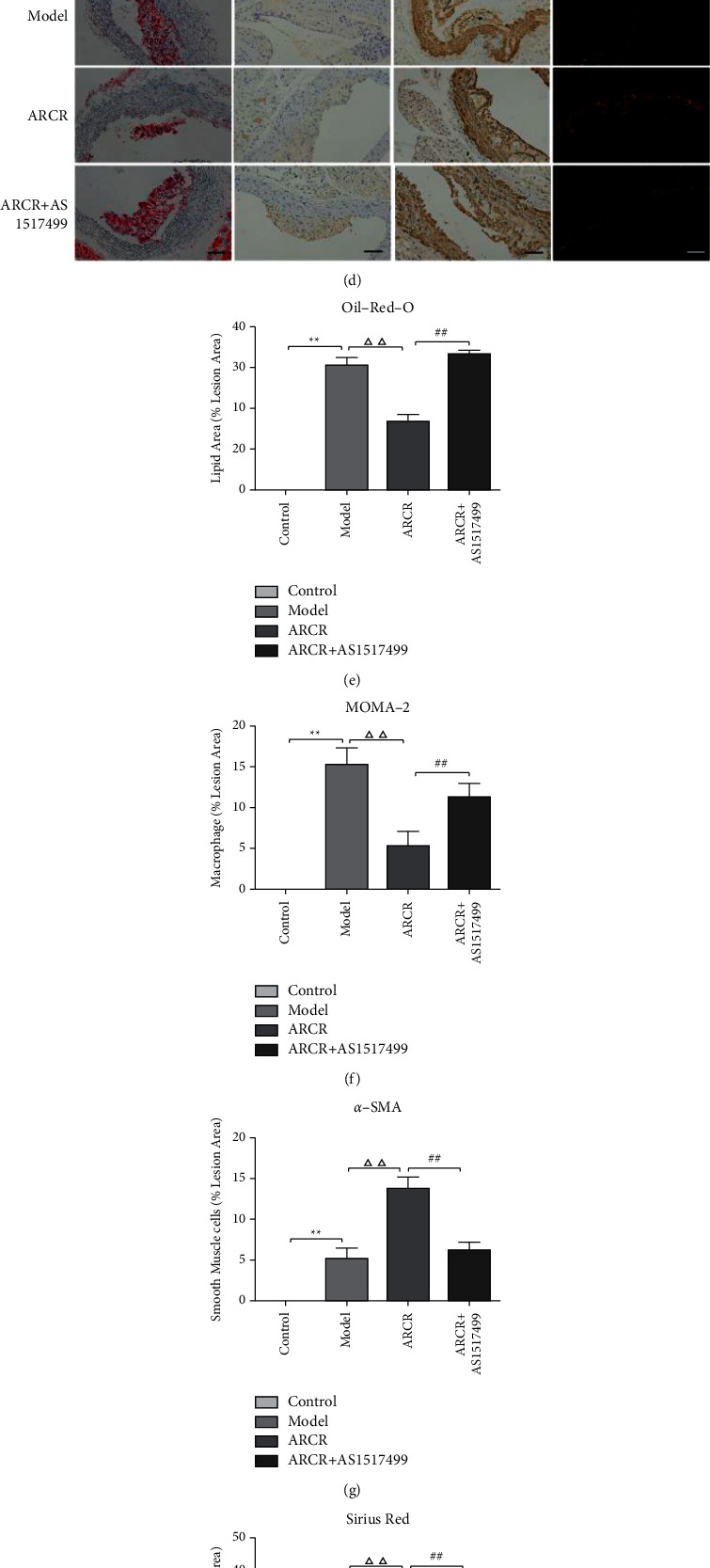
The ARCR herb pair attenuated atherosclerosis via a STAT6-dependent pathway. (a) Body weight, (b) TC, (c) TG, (d) oil red O staining of lipids, immunohistochemical staining of MOMA-2 (macrophage marker) and smooth muscle cells, and Sirius red staining of collagen. (e) Lipid area of oil red O staining. (f-g) Macrophages and smooth muscle cells were assessed using immunohistochemical staining. (h) Collagen of Sirius red staining. (i) Vulnerability index of plaque. (j) ELISA for MMP-9 expression. The data are presented as the means ± SD, *n* = 10, scale bars: 50 *μ*m. ^*∗*^*P* < 0.05, ^*∗∗*^*P* < 0.01 vs. the control group; ^Δ^*P* < 0.05, ^ΔΔ^*P* < 0.01 vs. the model group; ^#^*P* < 0.05, ^##^*P* < 0.01 vs. ARCR group.

As shown in Figure 6, the ARCR + AS1517499 group had increased body weight (*P* < 0.05, Figure 6(a)), TC and TG levels (*P* < 0.01, Figures 6(b) and 6(c)), decreased plaque stability (*P* < 0.01, Figures 6(d)–6(i)), and increased MMP-9 (*P* < 0.01, Figure 6(j)) compared to the ARCR group, which indicates that the inhibitory effect of the ARCR herb pair on atherosclerosis is related to activation of the STAT6-dependent pathway.

### 3.6. *Astragali Radix-Coptis Rhizoma* Herb Pair Regulated M1/M2 and Th1/Th2 Differentiation via a STAT6-Dependent Pathway

**Figure 7:**
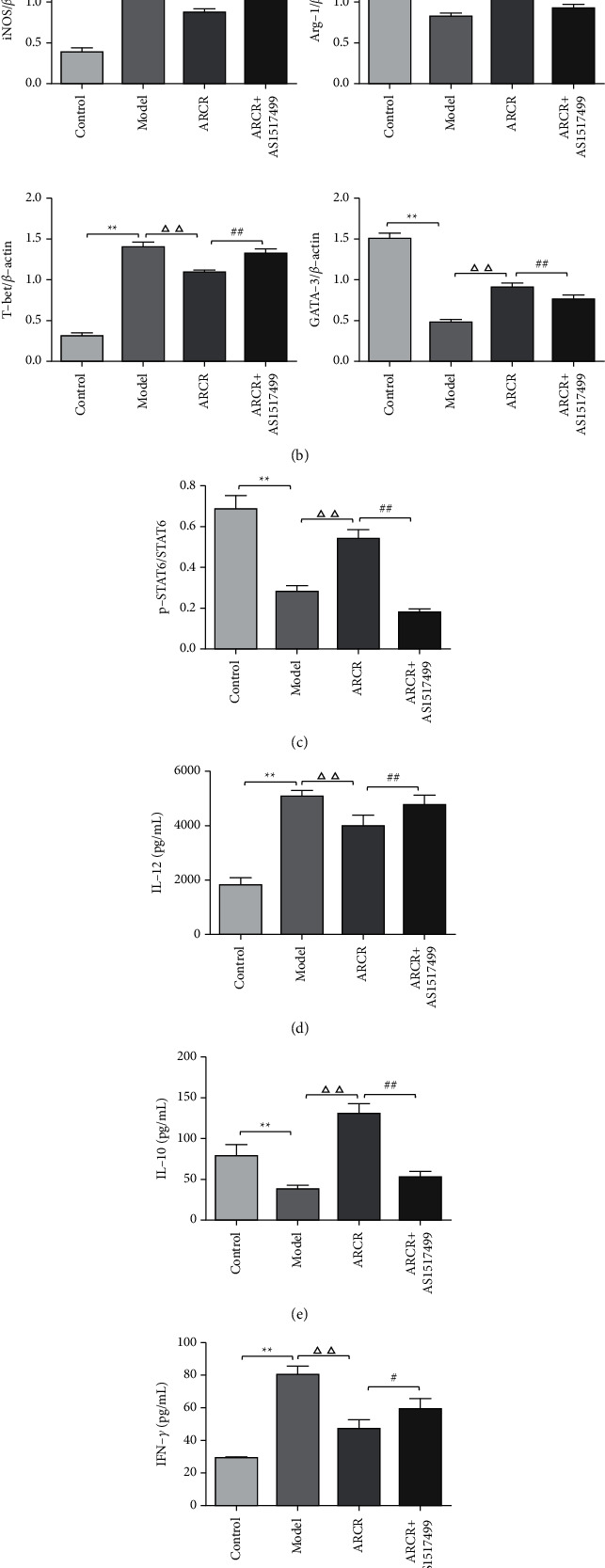
The ARCR herb pair promoted the phosphorylation of STAT6 and regulated inflammatory cytokines. (a) Representative western blot of iNOS, Arg-1, T-bet, GATA-3 and p-STAT6. (b) iNOS, Arg-1, T-bet and GATA-3 expression relative to the *β*-actin level. (c) p-STAT6 expression relative to the STAT6 level. (d) ELISA for the expression of proinflammatory cytokines IL-12 and (f) IFN-*γ* and the anti-inflammatory cytokines (e) IL-10 and (g) IL-4 in the supernatant. The data are presented as the means ± SD, *n* = 4. ^*∗*^*P* < 0.05, ^*∗∗*^*P* < 0.01 vs. the control group; ^Δ^*P* < 0.05, ^ΔΔ^*P* < 0.01 vs. the model group; ^#^*P* < 0.05, ^##^*P* < 0.01 vs. ARCR group.

**Figure 8:**
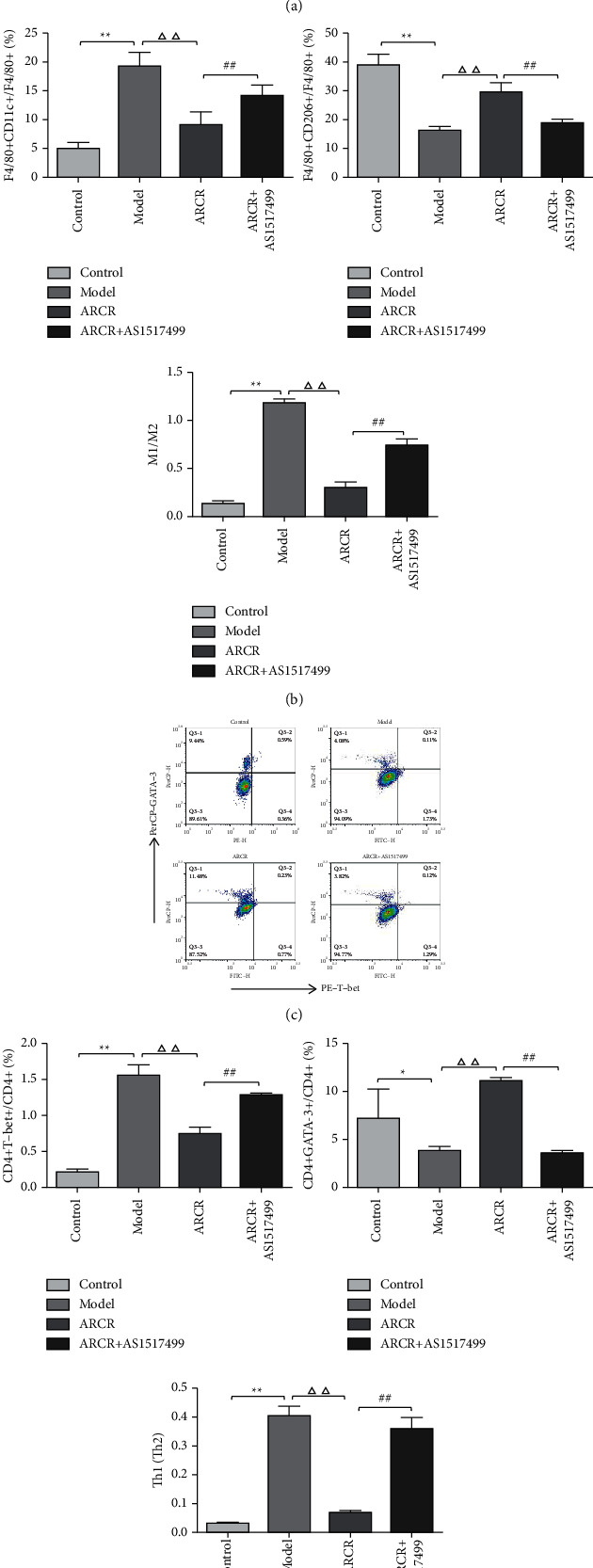
The ARCR herb pair regulated the M1/M2 and Th1/Th2 balance via a STAT6-dependent pathway. (a) The expression of F4/80 + CD11c+ and F4/80 + CD206+ cells was examined using flow cytometry. (b) Percentages of M1 and M2 macrophages and M1/M2 macrophages. (c) The expression of CD4 + CD25 + T-bet+ and CD4 + CD25 + GATA-3+ cells was examined. (d) Percentages of Th1 and Th2 cells and Th1/Th2. The data are presented as the means ± SD, *n* = 4. ^*∗*^*P* < 0.05, ^*∗∗*^*P* < 0.01 vs. the control group; ^Δ^*P* < 0.05, ^ΔΔ^*P* < 0.01 vs. the model group; ^#^*P* < 0.05, ^##^*P* < 0.01 vs. ARCR group.

**Figure 9:**
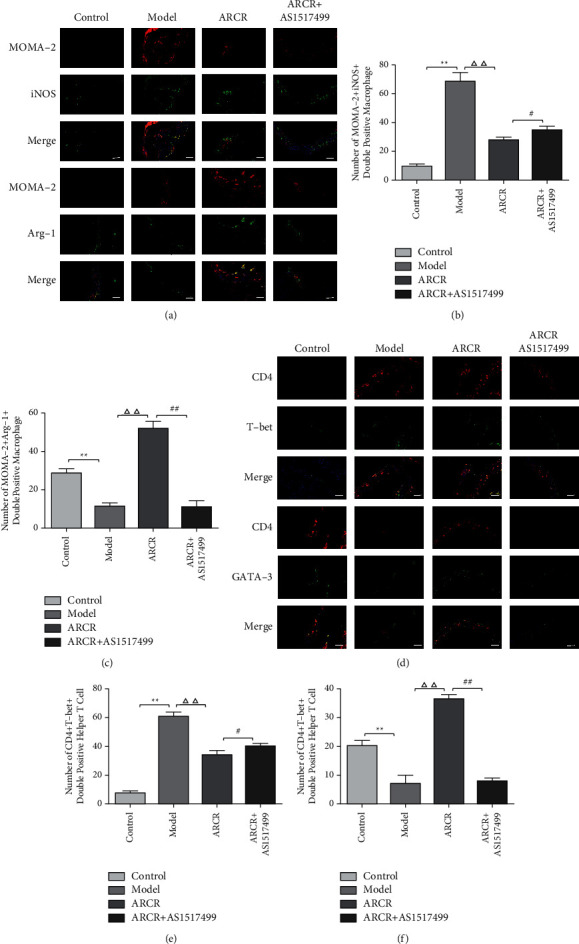
The ARCR herb pair regulated the M1/M2 and Th1/Th2 balance in atherosclerotic lesions via a STAT6-dependent pathway. (a) Representative images of MOMA-2 + iNOS+ and MOMA-2 + Arg-1+ macrophages. (b-c) Statistics of the number of MOMA-2 + iNOS+ and MOMA-2 + Arg-1+ macrophages in atherosclerotic lesions. (d) Representative images of CD4 + T-bet+ and CD4 + GATA-3+ cells. (e-f) Statistics of the number of CD4 + T-bet+ and CD4 + GATA-3+ cells in atherosclerotic lesions. The data are presented as the means ± SD, *n* = 3, scale bars: 50 *μ*m. ^*∗*^*P* < 0.05, ^*∗∗*^*P* < 0.01 vs. the control group; ^Δ^*P* < 0.05, ^ΔΔ^*P* < 0.01 vs. the model group; ^#^*P* < 0.05, ^##^*P* < 0.01 vs. ARCR group.

We further investigated whether the ARCR herb pair inhibited AS via STAT6-mediated immune cell polarization regulation. The results showed that the ARCR herb pair promoted the phosphorylation of STAT6 (*P* < 0.01, Figures 7(a) and 7(c)), and the STAT6 inhibitor (AS1517499) significantly inhibited the increase in Arg-1 and GATA-3 and the decrease in iNOS and T-bet induced by the ARCR herb pair (*P* < 0.01, Figures 7(a) and 7(b)). The proinflammatory factors IL-12 and IFN-*γ* in the ARCR + AS1517499 group increased significantly (*P* < 0.01, Figures 7(d) and 7(f)), and the anti-inflammatory factors IL-10 and IL-4 were significantly reduced (*P* < 0.01, Figures 7(e) and 7(g)). Flow cytometry analysis showed that the use of AS1517499 increased the number of M1 and Th1 cells. In contrast, it reduced the number of M2 and Th2 cells and inhibited the effect of the ARCR herb pair in regulating the M1/M2 and Th1/Th2 balance (Figure 8). The results of double immunofluorescence analysis were consistent with these results (Figures 9(a) and 9(d)). Compared to the ARCR group, the number of MOMA-2 + iNOS + double-positive macrophages (M1 macrophages) and CD4 + T-bet + double-positive cells (Th1 cells) in atherosclerotic lesions in the ARCR + AS1517499 group was significantly increased (*P* < 0.05, Figures 9(b) and 9(e)). The number of MOMA-2 + Arg-1+ double-positive macrophages (M2 macrophages) and CD4 + GATA-3+ double-positive cells (Th2 cells) was significantly decreased (*P* < 0.01, Figures 9(c) and 9(f)). These results suggested that ARCR herb pair treatment regulated M1/M2 and Th1/Th2 balance in atherosclerotic lesions via a STAT6-dependent pathway.

## 4. Discussion

The present study confirmed that the ARCR herb pair regulated the M1/M2 and Th1/Th2 balance and restricted the inflammatory response, which further contributed to the attenuation of AS in ApoE-/- mice. The effects of the ARCR herb pair on M1/M2 and Th1/Th2 balance were primarily achieved via increased STAT6 phosphorylation.

Many types of animal models are used in AS research. Apolipoprotein E knockout (ApoE-/-) mice are more mature models of atherosclerosis. The plasma cholesterol level of ApoE-/- mice is 4 to 5 times normal mice, and atherosclerotic lesions occur spontaneously under a normal diet. The pathological process is similar to humans [21, 22]. Sex also has an important influence on the development of AS. Most studies showed that women of childbearing age were less likely to develop atherosclerosis and cardiovascular diseases than men, and postmenopausal women had the same risk of developing diseases as age-matched men [23]. A large number of studies showed that endogenous oestrogen in mice had an antiatherosclerotic effect, and oestrogen replacement therapy played a role in relieving pathological changes [24]. Therefore, male mice were used to construct a model of atherosclerosis in this experiment.

AS is an important pathological basis of coronary artery disease, cerebrovascular disease, and peripheral artery disease, and it is one of the main causes of death worldwide [25]. The current main method to improve AS and its complications is the reduction of LDL [26]. However, the incidence of cardiovascular adverse events is not significantly reduced [27]. Inflammatory mechanisms may partially explain these differences. As shown in the Jupiter trial, statin therapy reduced the incidence of cardiovascular events by reducing LDL cholesterol and hs-CRP and may improve the functional capacity of HDL to inhibit the oxidation of low-density lipoprotein to exert anti-inflammatory effects [28, 29]. With the in-depth study of the immune and inflammatory mechanisms of AS, immune regulation has become an important target for improving AS.

Abnormal lipid metabolism and inflammation are the main risk factors for the occurrence and development of AS. Lipid infiltration theory and inflammation theory play dominant role in the pathogenesis of AS. Lipid metabolism disorders and inflammatory reactions are most important for the occurrence and development of AS. Inflammation leads to disorder of cellular lipid metabolism, which may lead to or aggravate the inflammatory response. Based on its pathogenesis, the effective prevention and treatment of AS must regulate lipid metabolism and inhibit the expression of proinflammatory factors or promote the expression of anti-inflammatory factors.

Dyslipidemia and many risk factors are very important independent factors. In the body's lipid metabolism process, hyperlipidemia promotes the entry of lipids into the arterial wall and stimulates a large number of immune cells to gather and form foam cells. These cells thicken the vascular intima and lead to AS. The results of the present study showed that ARCR improved lipid metabolism in model mice, including reducing TC and TG. The results of Oil Red O, immunohistochemistry, and Sirius Red staining showed that ARCR effectively reduced plaques in the aortas of mice and had a pharmacological effect on the formation and stabilization of plaques.

Inflammation plays a key role in the occurrence and development of AS. The main manifestations in the early stage of AS are endothelial damage and abnormal lipid metabolism. Monocytes and lymphocytes are also recruited, and the inflammatory response begins. Many immune cells and cytokines participate in this process, such as monocytes-macrophages, lymphocytes (T and B cells), dendritic cells (DCs), vascular smooth muscle cells (VSMCs), IL, and tumor necrosis factor-*α* (TNF-*α*) [30]. VSMCs and endothelial cells secrete a large amount of macrophage colony stimulating factor (M-CSF) and granulocyte-macrophage colony stimulating factor (GM-CSF), which induce monocytes to differentiate into macrophages and bind to oxidized LDL particles (ox-LDL) to form foam cells, which marks the rebirth of AS plaques. Foam cells continue to secrete inflammatory cytokines, reactive oxygen species, and other mediators, and plaques continue to develop and mature due to cell apoptosis. More inflammatory cytokines enter the blood vessel wall in the late stage of AS, which aggravates local inflammation and the secretion of MMPs to degrade collagen fibers in the extracellular matrix of plaques. This degradation creates the thin plaque fibrous caps and promotes rupturing, which lead to thrombosis and subsequent thrombotic events.

The balanced polarization of M1/M2 macrophages and Th1/Th2 lymphocytes determines the development of AS inflammatory lesions. Macrophages are the main participants in the AS inflammatory response [31]. Under different inducing factors, macrophages are polarized into two different subtypes: classically activated M1 type (proinflammatory) and alternatively activated M2 type (anti-inflammatory). M1 macrophages are polarized by lipopolysaccharide (LPS), IFN-*γ*, GM-CSF, and so on and produce proinflammatory cytokines, such as IL-12, which promote inflammation and the development and rupture of AS plaques. In contrast, monocyte-macrophages may polarize to the M2 type under the induction of IL-4, IL-13, M-CSF, and other factors and secrete anti-inflammatory cytokines, such as IL-10 and transforming growth factor-*β* (TGF-*β*), which play a role in inhibiting inflammation and repairing damaged tissues. The balance of proinflammatory/anti-inflammatory cell polarization affects the development of plaques. Previous studies showed that the ratio of M1 to M2 was different at different stages of AS and different locations in AS plaques [32, 33]. M2-type infiltration is the main cause in the early stage of AS plaque injury, and the plaque tends to be stable. M1-type infiltration is large in the period of plaque rupture, and the secretion of inflammatory factors increases. M1-type macrophages are found in the most unstable plaque shoulders, which increases the formation of necrotic cores and plaque fragility, and the number of M1 and M2 macrophages in the fibrous cap is equal. Naïve CD4+ T cells in the adaptive immune system differentiate into various cell subgroups to participate in the AS immune response. Representative Th1 cells play a role in promoting AS [34]. Th1 cells produce IFN-*γ*, which promotes the formation of foam cells and accelerates the development of plaques. Th2 differentiation produces anti-inflammatory factors, such as IL-4, IL-5, and IL-13. The increase in the number of peripheral circulating Th2 cells is related to a decrease in AS plaques [35]. The decrease in AS is related to the decrease in the Th1/Th2 ratio [36]. The Th1/Th2 balance may be targeted in AS prevention and treatment research [37]. Our research first found that the ARCR herb pair slowed the development of AS in ApoE-/- mice, stabilized plaques, and regulated the balance of serum inflammatory factors. Our results confirmed that the ARCR herb pair improved AS by inhibiting inflammation. The model group showed that M1 and Th1 cells increased in ApoE-/- mice, while M2 and Th2 cells decreased, accompanied by aggravation of inflammation. The treatment results of the ARCR herb pair also correspond to the abovementioned theory. Flow cytometry showed that the ARCR herb pair regulated the balance of the number of cells. Western blot and immunofluorescence results indicated that the ARCR herb pair promoted the polarization of M1 macrophages to M2 and the polarization of Th1 lymphocytes to Th2 to balance proinflammatory/anti-inflammatory responses.

According to traditional Chinese medicine (TCM) theory, *Astragali Radix* could reinforce qi and invigorate vitality, and *Coptis Rhizoma* could clear heat and detoxify, and they are commonly used as herb pair. In some famous TCM prescriptions such as Dangguiliuhuang Tang, and some experience of prestigious Chinese physicians, *Astragali Radix* is the “Sovereign” herb and *Coptis Rhizoma* is “Minister” herb, and they complement each other and work together to replenish qi and detoxify. Some network pharmacological studies showed that the combination of ARCR had multicomponent, multitarget, and multichannel effects on the treatment of coronary heart disease and diabetes, and the main active ingredients of this herb pair were quercetin, kaempferol, berberine, (R)-hydroberberine, and so on. Among them, quercetin was a common component of ARCR [38, 39]. Researchers have widely reported the beneficial physiological roles of quercetin, which has antioxidative, anti-inflammatory, and antifibrotic effects on atherosclerosis [40, 41]. These provide a theoretical basis for this herb pair as having a combination of anti-AS effects. However, the mechanisms of the combined effects are not verified. In this work, the results showed that the weight loss of mice in ARCR group was more obvious than that of AR or CR group. And ARCR herb pair was better than AR or CR in improving blood lipids and balancing proinflammatory/anti-inflammatory factors. ARCR herb pair significantly improved the vulnerability of aortic plaque in AS mice, but there was no statistically significant difference between AR or CR in reducing MMP-9. Although the flow cytometry showed that ARCR, AR, and CR groups have no significant differences in M1, M2 macrophages and Th2 lymphocyte content, but the ratio of M1/M2 and Th1/Th2 showed that ARCR herb pair could better regulate immune balance. Therefore, the above discussion and results may fully clarify that ARCR herb pair could produce combination effects on improving AS and regulating immune balance.

We further explored the underlying mechanisms of the ARCR herb pair regulation of the M1/M2 and Th1/Th2 balance. The pathway focuses on STAT6, which is differentially expressed in AS and is a potential new target in human atherosclerotic diseases [42]. Some drugs alleviate the progression of atherosclerosis via activation of STAT6 [43]. STAT6 signal activation promoted the differentiation of macrophages to the M2 type, and it induced T lymphocytes to differentiate to the Th2 type and inhibit Th1-type cells. Our study found that the ARCR herb pair effectively promoted STAT6 phosphorylation and regulated the M1/M2 and Th1/Th2 balance to attenuate AS. This effect was abrogated by the addition of a STAT6 inhibitor. Therefore, we concluded that the ARCR herb pair regulated the M1/M2 and Th1/Th2 balance via a STAT6-dependent pathway.

## 5. Conclusion

The ARCR herb pair regulated blood lipid metabolism and attenuated atherosclerosis in ApoE-/- mice by regulating the M1/M2 and Th1/Th2 immune balance and activating the STAT6 signaling pathway. These findings provide new experimental evidence for the use of ARCR herbs to prevent and treat AS and provide new strategies and methods to prevent and treat AS by regulating the balance of proinflammatory/anti-inflammatory responses.

## Figures and Tables

**Table 1:** Animal groups and drug administration.

Group	Treatment	Drug administration (i.g.)
Control	ordinary rodent chow	Normal saline
Model	high-fat diet	Normal saline
Simvastatin (simva)	high-fat diet	Simvastatin (5 mg/kg/d)
*Astragali radix* (AR)	high-fat diet	*Astragali radix* (3.9 g/kg/d)
*Coptis rhizoma* (CR)	high-fat diet	*Coptis rhizoma* (1.3 g/kg/d)
*Astragali radix*–*Coptis rhizoma* (ARCR)	high-fat diet	ARCR (5.2 g/kg/d)
ARCR + AS1517499	high-fat diet and AS1517499 (i.p.)	ARCR (5.2 g/kg/d)

Note: i.g.: intragastric administration; i.p.: intraperitoneal administration.

## Data Availability

The datasets used and/or analyzed during the current study are available from the corresponding author upon reasonable request.
